# Early Modulation of Circulating MicroRNAs Levels in HER2-Positive Breast Cancer Patients Treated with Trastuzumab-Based Neoadjuvant Therapy

**DOI:** 10.3390/ijms21041386

**Published:** 2020-02-18

**Authors:** Serena Di Cosimo, Valentina Appierto, Sara Pizzamiglio, Marco Silvestri, José Baselga, Martine Piccart, Jens Huober, Miguel Izquierdo, Lorena de la Pena, Florentine S. Hilbers, Evandro de Azambuja, Michael Untch, Lajos Pusztai, Kathleen Pritchard, Paolo Nuciforo, Anne Vincent-Salomon, Fraser Symmans, Giovanni Apolone, Filippo G. de Braud, Marilena V. Iorio, Paolo Verderio, Maria Grazia Daidone

**Affiliations:** 1 Biomarker Unit, Department of Applied Research and Technological Development, Fondazione IRCCS Istituto Nazionale dei Tumori, 20100 Milan, Italy; Serena.DiCosimo@istitutotumori.mi.it (S.D.C.); Valentina.Appierto@istitutotumori.mi.it (V.A.); Marco.Silvestri@istitutotumori.mi.it (M.S.); mariagrazia.daidone@istitutotumori.mi.it (M.G.D.); 2 Bioinformatics and Biostatistics Unit, Department of Applied Research and Technological Development, Fondazione IRCCS Istituto Nazionale dei Tumori, 20100 Milan, Italy; paolo.verderio@istitutotumori.mi.it; 3 Vall D’Hebron Institute of Oncology, 08035 Barcelona, Spain; jbaselga@vhio.net (J.B.); pnuciforo@vhio.net (P.N.); 4 Department of Medical Oncology, Institut Jules Bordet and l’Université Libre de Bruxelles (U.L.B), 1000 Brussels, Belgium; martine.piccart@bordet.be (M.P.); evandro.azambuja@bordet.be (E.d.A.); 5 Department of Obstetrics and Gynecology, University of Ulm, 89081 Ulm, Germany; Jens.Huober@uniklinik-ulm.de; 6 Novartis Pharmaceutical, 4002 Basel, Switzerland; miguel.izquierdo@novartis.com; 7 SOLTI—Breast Cancer Research Group, 08035 Barcelona, Spain; lorena.delapena@gruposolti.org; 8 Breast International Group (BIG)-aisbl, 1000 Bruxelles, Belgium; Florentine.Hilbers@bigagainstbc.org; 9 Department of Gynecology and Obstetrics, Helios Klinikum Berlin-Buch, 13125 Berlin, Germany; michael.untch@helios-kliniken.de; 10 Yale Cancer Center, Yale School of Medicine, New Haven, CT 06511, USA; laurene.goode-gade@yale.edu; 11 Division of Medical Oncology, Department of Medicine, Sunnybrook Health Sciences Centre, Toronto, M4N 3M5 ON, Canada; sabina.trebinjac@sunnybrook.ca; 12 Groupe d’étude des facteurs pronostiques immunohistochimiques dans le cancer du sein, 75013 Unicancer, France; anne.salomon@curie.fr; 13 Department of Pathology, The UT M.D. Anderson Cancer Center, Houston, TX 77030, USA; fsymmans@mdanderson.org; 14 Scientific Directorate, Fondazione IRCCS Istituto Nazionale dei Tumori, 20100 Milan, Italy; giovanni.apolone@istitutotumori.mi.it; 15 Department of Oncology, Fondazione IRCCS Istituto Nazionale dei Tumori, 20100 Milan, Italy; filippo.debraud@istitutotumori.mi.it; 16 Molecular Targeting Unit, Department of Research, Fondazione IRCCS Istituto Nazionale dei Tumori, 20100 Milan, Italy; Marilena.Iorio@istitutotumori.mi.it

**Keywords:** circulating microRNAs, biomarkers, HER2, breast cancer, trastuzumab, ct-miR-148a-3p

## Abstract

Circulating microRNA (ct-miRNAs) are able to identify patients with differential response to HER2-targeted therapy. However, their dynamics are largely unknown. We assessed 752 miRNAs from 52 NeoALTTO patients with plasma pairs prior and two weeks after trastuzumab. Increased levels of ct-miR-148a-3p and ct-miR-374a-5p were significantly associated with pathological complete response (pCR) (*p* = 0.008 and 0.048, respectively). At a threshold ≥ the upper limit of the 95%CI of the mean difference, pCR resulted 45% (95%CI 24%–68%), and 44% (95%CI 22%–69%) for ct-miR-148a-3p and ct-miR-374a-5p, respectively. Notably, ct-miR-148a-3p retained its predictive value (OR 3.42, 95%CI 1.23–9.46, *p* = 0.018) in bivariate analysis along with estrogen receptor status. Combined information from ct-miR-148a-3p and ct-miR140-5p, which we previously reported to identify trastuzumab-responsive patients, resulted in greater predictive capability over each other, with pCR of 54% (95%CI 25%–81%) and 0% (95%CI 0%–31%) in ct-miR-148a/ct-miR-140-5p high/present and low/absent, respectively. GO and KEGG analyses showed common enriched terms between the targets of these ct-miRNAs, including cell metabolism regulation, AMPK and MAPK signaling, and HCC progression. In conclusion, early modulated ct-miR-148-3p may inform on the functional processes underlying treatment response, integrate the information from already available predictive biomarkers, and identify patients likely to respond to single agent trastuzumab-based neoadjuvant therapy.

## 1. Introduction

Neoadjuvant therapy (NAT) is considered the standard of care in high-risk early breast cancer (BC) with an indication for chemotherapy, such as in the HER2-positive BC subtype. NAT can achieve tumor down-staging before surgery. In the context of HER2-positive BC, the concurrent or sequential addition of HER2-targeted agents to chemotherapy-based NAT improves the rate of pathological complete response (pCR) [1]. As the achievement of pCR translates into favorable prognosis, research is increasingly focusing on the identification of biomarkers to closely monitor response to NAT, and to promptly adapt treatment to patient individualized risk [2,3,4,5]. Most of these studies used primary tumor tissue samples, although liquid biopsy, as a source for tumor-derived information, offers the advantages of being non-invasive and providing a dynamic “snapshot” of the entire tumor burden at specific time points, which allows identification of changes reflecting individual response to treatment [6,7].

Plasma or serum microRNAs (miRNAs) have emerged as promising liquid biopsy biomarkers due to their stability under storage, easy handling conditions, and emerging expression signatures, which are associated with prognosis and treatment response [8,9]. Circulating miRNAs (ct-miRNAs) exist as cell-free, protein-bound molecules that are released by apoptotic and/or necrotic cells into the blood, but are also actively released in exosomes [10,11]. High levels of ct-miR-21, ct-miR-4734, and ct-miR-150-5p have been associated with poor clinical outcome in HER2-positive non metastatic BC patients treated with NAT, or adjuvant therapy ± trastuzumab [12,13]. ct-miR-210 should be added to this unfavorable prognostic list because, while initially considered to be a predictive factor of response to trastuzumab, it was later shown to be associated with decreased event-free survival (EFS) [14]. More recently, our group reported four different treatment- and time-specific ct-miRNA signatures able to identify HER2 positive BC patients with differential response in terms of pCR to neoadjuvant trastuzumab, lapatinib and their combination followed by paclitaxel [15].

Here, we analyzed data from plasma samples prospectively collected from HER2-positive BC patients receiving trastuzumab-based NAT within the NeoALTTO trial [16] to identify changes in ct-miRNAs levels during the first two weeks of treatment, and to explore any association of early ct-miRNA dynamics with final patient response to NAT and clinical outcome.

## 2. Results

### 2.1. Patient Characteristics and Response to Therapy

Overall, 52 patients treated with trastuzumab and with matched T0 and T1 complete ct-miRNA profile were included in this analysis. Table 1 reports their clinical and pathological characteristics. Median patient age was 48.5 (range 25–70) years. The majority of patients were diagnosed with primary tumor size ≤ 5 cm (63%) and N0/1 (83%). Breast pCR rate was 31%. The distribution of patient characteristics of this sub-study was superimposable to those of the entire NeoALTTO patient population [16].

### 2.2. ct-miRNA Level Changes during Treatment with Trastuzumab

**Figure 1:**
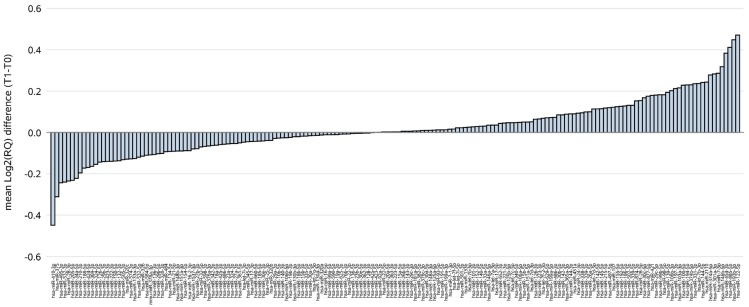
Waterfall plot of the mean difference between week 2 (T1) and baseline sample (T0) for the 177 considered ct-miRNAs. ct-miRNAs on the x-axis are sorted according to the mean change between T1 and T0 samples expressed as log_2_RQ.

Among a total of 752 screened human miRNAs in plasma samples, 177 ct-miRNAs, i.e., those detected in at least 10 patients with pCR, were considered in the statistical analysis. All ct-miRNAs were analyzed at baseline (T0, i.e., before commencing treatment) and after just 2 weeks of treatment (T1, i.e., after two doses of trastuzumab). Hence, our findings refer to a very early assessment of ct-miRNAs profile performed 4 months in advance to final surgery, when pCR was finally evaluated. The waterfall plot of each ct-miRNA in terms of mean difference between T1 and T0 expressed as log_2_RQ (Figure 1) showed that ct-miR-410-3p and ct-miR-122-5p were those with the broadest fold changes (FC, 2^[log2(RQ)T1 − log2(RQ)T0]), in terms of levels reduction (FC = 0.73), and increase (FC = 1.38), respectively.

Figure S1 reports the box plot of the distribution of the log_2_ relative amount of each ct-miRNA at T0 and T1. Notably, the ranges were broader in post-treatment (T1) compared with baseline (T0) samples. The ct-miRNAs with the wider range of log2 expression were ct-miR-205-5p (from −13.17 to −3.09) at baseline, and ct-miR410-3p (from −18.96 to −3.31) in post-treatment samples.

### 2.3. ct-miRNA Changes Are Associated with pCR

**Figure 2:**
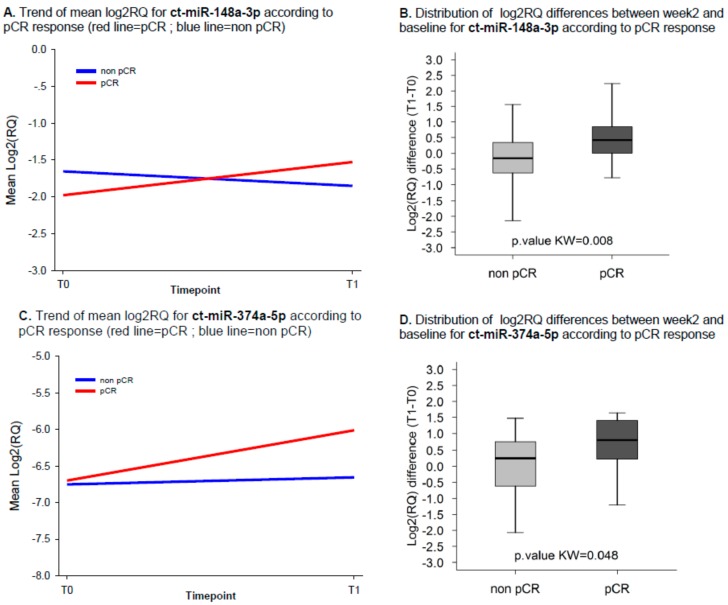
Trend of mean log_2_RQ and distribution of the difference between week 2 and baseline according to pCR for ct-miR-148a-3p and ct-miR-374a-5p. Panel (**A**) and (**C**) report the trend of mean log_2_RQ for ct-mR-148a-3p and ct-miR-374a-5p according to pathological complete response (pCR), respectively. Panel (**B**) and (**D**) report the distribution of the difference between log2RQ of each ct-miRNA at T1 and T0 according to pCR. Red line indicates patients with pCR and blue line indicates patients without pCR. Panels (**B**) and (**D**) report the distribution of log2RQ difference between week 2 and baseline for ct-mR-148a-3p and ct-miR-374a-5p according to pCR, respectively. *p*-Value of the Kruskal-Wallis test for the comparisons of log2RQ difference between patients with and without pCR is reported.

Only two ct-miRNAs, i.e., ct-miR-148a-3p and ct-miR-374a-5p, showed changes in their levels significantly associated with pCR (Kw test *p*-values of 0.008 and 0.048, respectively). ct-miR-148a-3p and ct-miR-374a-5p were detected in 96% and 75% of patients with both T0 and T1 samples, respectively. Overall, the expression levels of both ct-miR-148a-3p and ct-miR-374a-5p increased during treatment in patients attaining pCR (Figure 2A,C). Box plots in Figure 2B,D report the distribution of the difference between log2RQ of each ct-miRNA at T1 and T0 according to pCR. The median change level for both ct-miRNAs increased in patients achieving pCR as compared to those with residual disease at surgery. Specifically, for ct-miR-148a-3p we observed a median FC of 1.34 and 0.90 in patients with and without pCR, respectively. Similarly, for ct-miR-374a-5p median FCs of 1.73 and 1.19 were observed in the two groups. When we dichotomized the study patient population according to the upper limit of the 95% CI (UL), i.e., classifying patients above or equal to this threshold in the category “high changes”, the pCR rate was 45% (95% exact CI 24%–68%) for miR-148a-3p (Figure S2A) and of 44% (95% exact CI 22%–69%) for miR-374a-5p (Figure S2A). On the other hand, by classifying patients below the UL threshold in the category “low/intermediate changes”, the pCR rate was 21% (95% exact CI 8%–41%) for miR-148a-3p and of 19% (95% exact CI 5%–42%) for miR-374a-5p.

### 2.4. Univariate and Multivariate Logistic Models

Considering the changes as a continuous variable, among the 177 ct-miRNAs, only ct-miR-148a-3p resulted in being significantly associated with pCR in uni- and multivariate logistic regression models (including ER status), whereas borderline *p*-values of 0.077 and 0.073 were observed for ct-miR-374a-5p in uni- and multivariate logistic regression models, respectively (Table 2). The predictive capability of ct-miR148a-3p was statistically significant with AUC values of 0.74 (95%CI: 0.59; 0.88). At a median follow-up of 6.7 years (inter quartile range 6.3–6.9), 16 events were observed in the study population. The log-rank test and the Hazard Ratios (HR) reported in Supplementary Table S1 indicated that neither miR148a-3p nor miR-374a-5p were associated with EFS.

### 2.5. Changes of ct-miR148a-3p Jointly Considered with the Evaluation of ct-miRNA140-5p at Week 2

By taking advantage of our previous work [15], that identified ct-miR140-5p as associated with both pCR and favorable prognosis after two weeks of treatment with trastuzumab, we performed an additional explorative analysis to address whether the changes of ct-miR-148a-3p provided overlapping or rather complementary information as compared to the levels of ct-miR 140-5p at week 2. To this end the set of 48 patients with both miRNAs was considered and for miR-148a-3p the identified UL was used as a threshold to classify patients whereas for ct-miR-140-5p patients were classified in the category “hight level” and “low level” according to the threshold previously reported [15]. By jointly considering both ct-miRs, patients with “high changes” of ct-miR-148a-3p and high values of ct-miR-140-5p (i.e., 13/48 cases, 27%) had a chance of pCR up to 54% (7/13, 95% exact CI 25%–81%); conversely, patients with “low/intermediate changes” of ct-miR-148a-3p and low values of ct-miR140-5p (i.e., 10/48 cases, 21%) had a 0% (0/10, 95% exact CI 0%–31%) chances of pCR. For the remaining patients with discordant classification between the two considered ct-miRs, had the pCR rate was 28% (7/25, 95% exact CI 12%–49%).

### 2.6. Association of miR-140-5p, miR 148a-3p and mir-374a-5p with Functional Related Pathways

**Figure 3:**
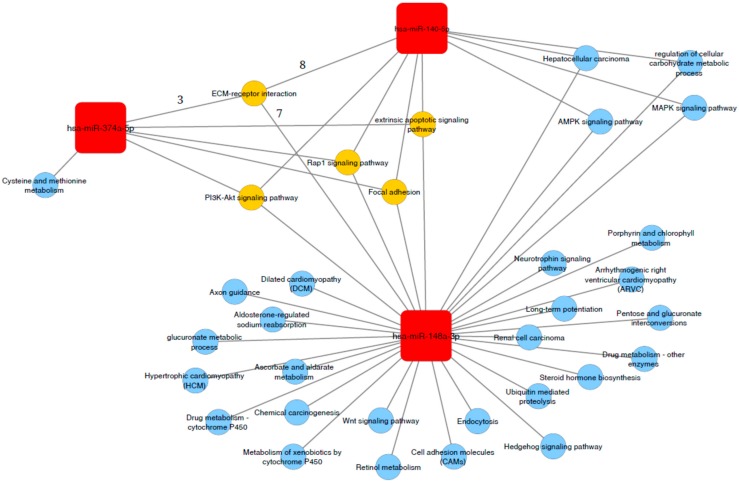
Functional interactions network analysis of ct-miR-148a-3p,ct-miR-140-5p, and ct-miR-374-a-5p. Specific (represented in blue) and common targeted signaling pathways (represented in yellow) of ct-microRNAs associated with pCR (i.e., miR 374a-5p, miR-148-3p and miR-140-5p, represented in red) obtained from GO and KEGG database analyses. The number of ct-miR-148a-3p,ct-miR-140-5p, and ct-miR-374-a-5p target genes related to ECM pathway are represented above the edges.

miRWalk analysis returned a total of 901 genes as predicted targets of miR-140-5p (n = 1554), ct-miR-148a-3p (n = 859), and miR-374a-5p (n = 47) identifying three functional modules strictly related to each miRNAs. In particular, the presence of multiple common genes between clusters delineate connected components, including ECM-receptor, PI3K-Akt, Rap1, focal adhesion and extrinsic apoptotic signaling (Figure 3). Moreover, genes related to miR 148a-3p and miR-140-5p were involved in pathways of regulation of cellular carbohydrate metabolic processes, AMPK, MAPK signaling and hepatocellular carcinoma progression. Interestingly, miR-140-5p had no significant private pathway, reinforcing the potential biological value of the network identified by the three ct-miRNAs all together.

## 3. Discussion

The value of circulating miRNAs as potential biomarkers for cancer patient management is not a novelty [17]. Nevertheless, data are few and conflicting in BC due to differences in study patient characteristics (stage of disease and type of treatment), source (plasma or serum) for profiling, and number of ct-miRNAs analyzed [18]. All this prompted us to perform a high throughput analysis of 752 miRNAs assays in prospectively collected plasma samples from HER2-positive BC patients treated within the large context of an international prospective randomized clinical study such as NeoALTTO [16]. The main focus of the present work was the identification of ct-miRNAs that were differentially expressed at week 2 as compared to baseline in patients who attained pCR compared to those with residual disease at surgery. After normalization, ct-miR 148a-3p and ct-miR-374a-5p turned out to be increased in the plasma samples of patients attaining pCR. Basal levels of both did not correlate with response to treatment but their dynamics predicted the ultimate clinical response, supporting the hypothesis that these two miRNAs are involved in the mechanism underpinning sensitivity to trastuzumab and might be used as early markers of response to treatment. Noteworthy, we found that increased miR-148a levels retained significance as an independent factor of pCR in multivariate analysis including ER status.

Under normal physiological conditions, miR-148a-3p and miR-374a-5p are expressed in various human tissues including heart, liver, bowel, thymus, pancreas, renal, prostate, placenta, uterus, testis, and the hematopoietic system (reviewed in [19,20]). Accumulating studies have shown that miR-148a-3p and miR-374a-5p are aberrantly expressed in tumor cells of various cancer type, including breast cancer. However, their precise biological functions and the mechanism(s) of regulation remain unsolved (Figure S3). As a tumor suppressor factor, miR-148a-3p was found down-regulated in BC cells and negatively associated with tumor grade and nodal involvement [21,22]. Consistently, in vitro studies reported that miR-148a over-expression inhibits BC cell proliferation through inhibition of MAPK/ERK signaling pathways by direct targeting of *ERBB3* genes [23]. Furthermore, miR-148a-3p can depress cell proliferation by dampening functional expression of IGF1R and IRS1, whose signaling is crucial in luminal breast cancer subtype [24,25]. In more recent studies, miR-148a-3p was found to reduce HLA-G and PD-L1 levels; therefore, its down-regulation contributes to a immunosuppressive tumor microenvironment [26,27]. The miR-148a-3p functions deserve to be framed in the complex crosstalk existing between estrogen and growth factor (GF) receptors. Numerous studies have indeed demonstrated that both estrogens and GFs stimulate the proliferation of steroid-dependent tumor cells, and that this interaction occurs at several levels. For example, estrogens are known to up-regulate IGF1R signaling by inducing cyclic AMP responses element-binding protein activation [28], and to trigger EGFR phosphorylation via Src [29]. On the other hand, HER2 signaling, especially through the HER2-HER3 heterodimer, can induce ER phosphorylation independently of the estrogen ligand, thus reducing the effectiveness of endocrine therapies [30].

Earlier research proved that the transcription factor EGR1 directly regulates the expression of miR148a-3p by binding to its promoter [31]. EGR1 is induced by treatment with anti-HER2 targeted therapies [32], including trastuzumab, and its expression is associated with favorable outcome in HER2 positive cancer models [33]. These evidence aided in better interpretation of our findings. We hypothesize that treatment with trastuzumab may up-regulate cellular miRNA-148a-3p via EGR1-dependent pathway. The consequence of sequential cycles, which induce a massive attack of tumor cells inducing apoptosis/necrosis, might increase the levels of ct-miR-148a-3p in responsive patients. On the other hand, for insensitive patients miR-148a-3p in the tumor tissue might be down-regulated through an unknown mechanism (i.e., DNA overmethylation), leading to reduced levels of plasma miR-148a-p3 after trastuzumab. This preliminary speculation needs verification in further research.

Another major finding of our study is the predictive value of ct-miRNA level changes beyond the detection of ct-miRNA-140-5p. Overall, we reported not only that increased levels of ct-miR-148a-3p could represent a valuable biomarker at net of ER status of pCR following two weeks of treatment with Trastuzumab, but also that if this ct-miRNA is jointly considered with ct-miRNA-140-5p, additional information can be provided. To our knowledge, this is the first attempt to report the results, although they were obtained from explorative analysis, based on biomarkers derived from liquid biopsy to track early response to treatment. As already discussed in our previous work, ct-miRNA-140-5p could be involved in the host response to therapy by reflecting the level of engagement of immune response [15], while ct-miR-148a-3p could be involved in the response of the primary tumor by reflecting the effect of treatment on tumor cells. Indeed, the GO and KEGG analysis showed no private pathways for miR-140-5p, but common pathways with other miRNAs involved in response to treatment. Among these pathways is PI3K/Akt, which is well known to be associated with anti-HER2 resistance.

At present, the definite molecular mechanisms of resistance to trastuzumab remain to be understood, though three major categories have been advocated including: steric effects, such as the structural mutation in HER2 protein; elevations of alternative tyrosine kinase receptors, such as insulin-like growth factor receptor (IGFR); or alterations in HER2 downstream signaling, as in the case of phosphatase and tensin homolog (PTEN) deficiency and/or PI3K/Akt constitutive activation [34]. Therefore, a comprehensive understanding of the specific mechanisms involved in differential response to treatment is needed for optimization of treatment at the patient level. Our integrated analysis of miRNA targeted genes networks showed that among predicted targeted genes, a substantial proportion were involved in cell metabolism and survival. Hence, differentially expressed circulating miRNAs might inform on the mechanisms mediating biological process soon after treatment with trastuzumab. We also assessed the associated signal pathways which were modulated by the differentially expressed mRNAs through GO and KEGG analysis. Among the enriched terms, those related with AMPK and MAPK signaling, and HCC processes were shared by all dysregulated ct-miRs during treatment with trastuzumab. Combining the function and pathway analysis of the predicted targets of microRNAs, we might conclude that treatment with trastuzumab exerts a selective pressure on ctmiRNA, which in turn can be an early informer on tumor response and eventually anticipate pathological response, as a result of drug-primary tumor and host interplay, as early as 2 weeks after starting treatment.

The ultimate goal of NeoALTTO was to prove that dual HER2 targeted therapy outperforms trastuzumab-based therapy in terms of pCR. Indeed pCR rate was significantly higher in the group given lapatinib and trastuzumab (78 of 152 patients [51.3%; 95% CI 43.1–59.5]) than in the group given trastuzumab alone (44 of 149 patients [29.5%; 22.4–37.5]; difference 21.1%, 9.1–34.2, *p* = 0.0001) [16].

The rate of pCR in patients with increased levels of ct-miR 148a-3p reached 45%, and increased to 54% in the presence of high levels of ct-miR 140-5p, approaching the results obtained by dual HER2-targeted therapy. Conversely, in cases of low levels of both markers, patients derived no benefit from treatment with trastuzumab. Though these data need further confirmation, it is reasonable that the development of ct-miRNA in HER2-positive BC could help to spare patients from intensive and expensive treatment.

Our study also has several limitations. The power of analysis was compromised because of the small capacity and number of events. Moreover, it should be admitted that the NeoALTTO regimen is not among the standard chemotherapy regimens for BC, especially for the presence of a biological therapeutic window of trastuzumab alone for the first 6 weeks of therapy. Importantly, the costs and variable accuracy of serial miRNA assay preclude its clinical implication, which might be overcome with technology improvement. Given that dynamics of plasma miRNAs might serve as a potential marker of chemo-sensitivity, its clinical relevance should be further validated in larger series of patients with BC.

In conclusion, this report reveals that early changes of ct-miR148-3a levels have the potential to inform on the core functional processes underlying trastuzumab response/resistance, integrate the information from already available “static” clinico-pathological and molecular factors, including ct-miR-140-4p, and finally to identify patients likely to respond to neoadjuvant trastuzumab-based therapy as early as two weeks after initiating treatment.

## 4. Materials and Methods

The current work is a retrospective analysis of the NeoALTTO prospective randomized clinical trial [16]. NeoALTTO was a multicenter phase III study on HER2 positive BC patients with primary tumor > 2 cm, which were randomly assigned to either lapatinib, trastuzumab, or their combination for 6 weeks, followed by 12 additional weeks with paclitaxel. Surgery was performed within four weeks from the last paclitaxel dose. After surgery, all patients received fluorouracil, epirubicin, and cyclophosphamide for three cycles and continued the same anti-HER2-targeted agent received prior to surgery to complete 52 weeks of treatment. The primary endpoint was pCR (i.e., absence of invasive tumor cells in the breast). The secondary endpoint EFS was defined as the time from randomization to first event. All patients enrolled were asked to sign the main study consent form, which included a non-specific clause for use of blood samples collected at baseline (T0), after two weeks of treatment (T1), prior to surgery (T2), and eventually at the time of relapse, for future biomarker research. The study complied with the Declaration of Helsinki. The Internal Review and Ethics Boards of Fondazione IRCCS Istituto Nazionale dei Tumori—Milan approved this ct-miRNA study.

### 4.1. Sample Collection and Processing

Starting from the ct-miRNA high-throughput profile of the NeoALTTO cases [15], we considered the suitable scope of the present analysis, which was to identify early ct-miRNA modulation associated with pCR, patients treated within the trastuzumab arm and with available complete 752 ct-miRNA profile on plasma pairs at T0 and T1 (Figure S4).

### 4.2. ct-miRNA Profiling Data Processing

Our procedures for plasma preparation and RNA isolation have been previously reported [15]. Reverse transcription and ct-miRNA profile was performed using the miRCURY LNA™Universal RT microRNA PCR system according to the Exiqon manufacturer’s instructions. A total of 752 ct-miRNAs were profiled using microRNA Ready-to-Use PCR, Human panel I+II in each sample. The amplification curves were analyzed using the Roche LC software for determination of quantification cycle (Cq) values. The relative quantity (RQ) of each ct-miRNA was calculated using the comparative threshold cycle method following the formula 2^- DCq where DCq = (Cq miRNA − Cq reference) [35], where the Cq reference was computed by considering the Cq average of all the detected miRNAs (global mean approach) [36,37]. The RQ of each miRNA, expressed in logarithmic scale (log2 RQ), was then used for the statistical analysis.

### 4.3. Statistical Analysis

For the statistical analyses we considered background filtered (BF) data as supplied by Exiqon (i.e., for assays that do not yield any signal on the negative control, the upper limit of detection was set to Cq = 37; otherwise it was set to 3 Cq lower than the Cq value of a negative control). The BF Cq values were then processed to obtain log2(RQ). The difference between log2(RQ) at T1 as compared to T0 (level change) was then analyzed in univariate manner to identify those ct-miRNAs whose change was statistically associated with pCR by resorting to the non-parametric Kruskal-Wallis test. In this selection step we considered as potentially relevant only those ct-miRNAs detected in at least 10 patients with pCR. The strength of association between the selected miRNAs and pCR was evaluated by resorting to univariate and multivariate logistic regression models with clinico-pathological variables, including Estrogen Receptor (ER) status. The Area Under the Receiver Operating Characteristic (ROC) Curve (AUC) and its 95% confidence interval (CI) were calculated to estimate the predictive capability of each model. For miRNAs of interest, the 95% CI of the mean difference between log2(RQ) at T1 versus T0 were computed and the upper limit was used as a threshold to classify patients in high versus low/intermediate changes categories. Within each category the 95% CI of the proportion of responders was computed by resorting to the exact binomial method. Finally, the prognostic role on EFS of each miRNA of interest was investigated using a Cox regression model. The patterns of EFS were estimated using the Kaplan–Meier method and compared using log-rank test. All statistical analyses were carried out with the SAS (Version 9.2.; SAS Institute, Inc., Cary, NC) and R software by adopting a significance level of α = 0.05. Prediction of target site of ct-miRNA(s) of interest was performed using miRWalk 3.0 [38]. Functional enrichment of ct-miRNA targeted genes for Gene Ontology (GO) biological process terms and KEGG pathways was performed using the ClusterProfiler Bioconductor package, and a false discovery rate (FDR) < 0.05.

## Figures and Tables

**Table 1:** Clinico-pathological characteristics of study population.

Variable	Characteristics	N	%
Age (years)	median (range)	48.5 (25;70)	
ER status	Positive	26	50
	Negative	26	50
Nodal status	N0/1	43	83
	N2	9	17
Tumor size	≤5 cm	33	63
	>5 cm	19	36
pCR	No	36	69
	Yes	16	31
Planned surgery	Conservative	12	23
	Not Conservative	40	77

**Table 2:** Association between changes in ct-miRNA levels and pathological complete response (pCR): univariate and multivariate logistic regression model by considering the Difference Log2RQ (week2-baseline) as continuous variables.

Difference Log2RQ (Week2-Baseline)	Univariate		Multivariate *	
	OR (95%CI)	*p*-Value	OR (95%CI)	*p*-Value
**ct-miR-148a-3p**	**3.42 (1.24;9.46)**	**0.018**	**3.42 (1.23;9.46)**	**0.018**
ct-miR-374a-5p	2.24 (0.92;5.48)	0.077	2.31 (0.93;5.78)	0.073

The bold values correspond to statistically significant odds ratio (OR) at alpha level of 0.05; CI, confidence interval; * multivariate model including estrogen receptor status.

## Supplementary Materials

Supplementary materials can be found at https://www.mdpi.com/1422-0067/21/4/1386/s1.
