# Supplementary material for: Early Modulation of Circulating MicroRNAs Levels in HER2-Positive Breast Cancer Patients Treated with Trastuzumab-Based Neoadjuvant Therapy
Source: Int J Mol Sci. 2020 Feb 18;21(4):1386. doi: 10.3390/ijms21041386 (PMC7073028; doi:10.3390/ijms21041386)
Supplement: Supplementary file 1 [file ijms-21-01386-s001.zip › 17_02_2020/Suppl_figure_legends.docx]

**Figure S1 Distribution of log2 (RQ) for the 177 considered ct-microRNAs at baseline and week2**. Box-plots reflect the distribution of microRNA expression within each time point. Each box (blue= sample at baseline and red= samples at week2) indicates the 25th and 75th percentiles of the distribution. The horizontal line inside the box indicates the median, and the whiskers indicate the extreme values (min and max value).

**Figure S2 Distribution of log2 (RQ) difference between week2 and baseline sample for ct-miR148a-3p and ct-mir374a-5p.** The grey lines represent the upper and lower limit of the 95% confidence interval of the mean difference between log2(RQ) at week2 and baseline. The grey and black symbol represent responsive and non responsive patients, respectively.

**Figure S3 miRNAs 148a-3p and 374-5p are expressed in normal breast tissue, peripheral blood cells, and their malignant counterparts.** HER2 positive breast cancer are characterized by lower levels of miR-148a-3p than normal controls. miR-148a-3p expression in primary tumor samples was analyzed from TCGA publicly available data. miRNA-148a-3p expression was reduced in patients with hematopoietic malignancies as compared with peripheral blood of healthy donors, as assessed from data collected from the GSE51908 database. No significant difference was observed for miRNA-374-3p in both datasets. Statistical analysis was performed using t-test.

**Figure S4 Workflow of the study population.** Of the 60 patients randomized into the Trastuzumab arm with complete 752 miRNA profile, 52 had matched plasma samples at T0 and T1, and were finally considered in this NeoALTTO sub-study. Abbreviations: PCR, Polymerase Chain Reaction, T0: Baseline sample, T1: week2 sample.
