# Supplementary material for: Early Modulation of Circulating MicroRNAs Levels in HER2-Positive Breast Cancer Patients Treated with Trastuzumab-Based Neoadjuvant Therapy
Source: Int J Mol Sci. 2020 Feb 18;21(4):1386. doi: 10.3390/ijms21041386 (PMC7073028; doi:10.3390/ijms21041386)
Supplement: Supplementary file 1 [file ijms-21-01386-s001.zip › 17_02_2020/Tables_S1.docx]

**Table S1** Univariate Cox regression model for the association of ct-miRNA level changes and Event-Free Survival

| **Difference log2RQ (we2-bas)** | **HR** | **95% CI** | | **p value log Rank test*** | **n. of events** |
| --- | --- | --- | --- | --- | --- |
| **ct-miR-148a-3p (n=50)** | 0.75 | 0.41 | 1.38 | 0.57 | 16 |
| **ct-miR-374a-5p (n=39)** | 1.35 | 0.67 | 2.7 | 0.66 | 11 |

HR: Hazard ratio; CI: confidence interval

* Dichotomizing the difference log2RQ (week 2-baseline) of miR-148a-3p and 374a-5p by using as threshold the upper limit of the 95% confidence interval of the mean difference.
