# Supplementary figures and images for: Early Modulation of Circulating MicroRNAs Levels in HER2-Positive Breast Cancer Patients Treated with Trastuzumab-Based Neoadjuvant Therapy
Source: Int J Mol Sci. 2020 Feb 18;21(4):1386. doi: 10.3390/ijms21041386 (PMC7073028; doi:10.3390/ijms21041386)

**TCGA miRNA 148a-3p**

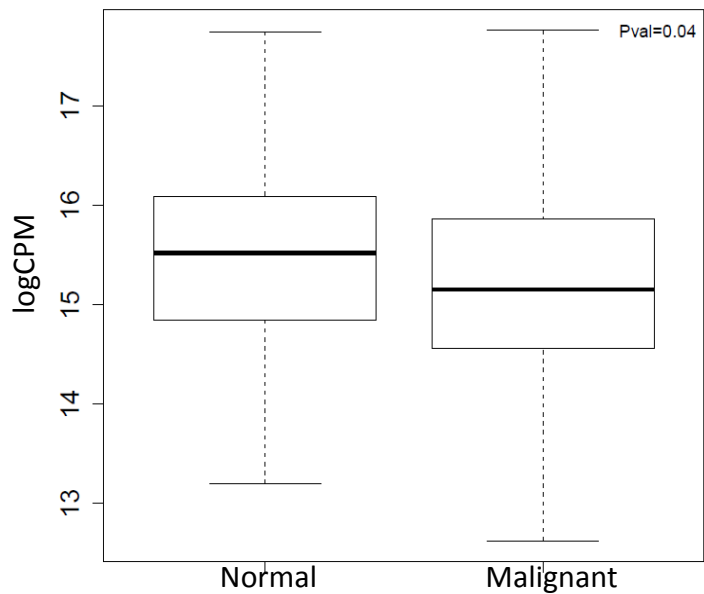

**GSE51908 miRNA 148a-3p**

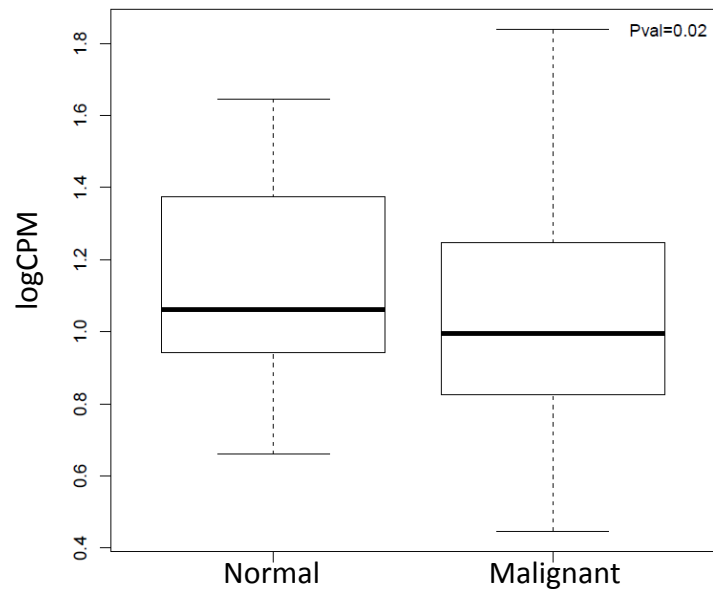

**TCGA miRNA 374a-5p**

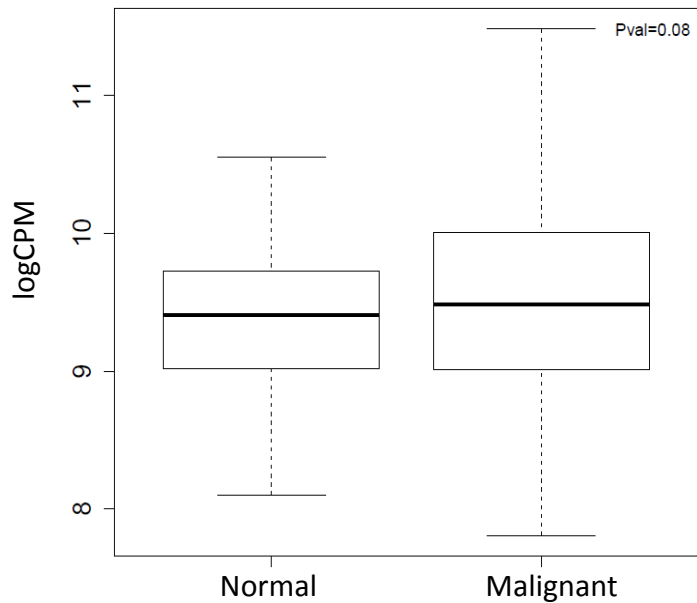

**GSE51908 miRNA 374a-5p**

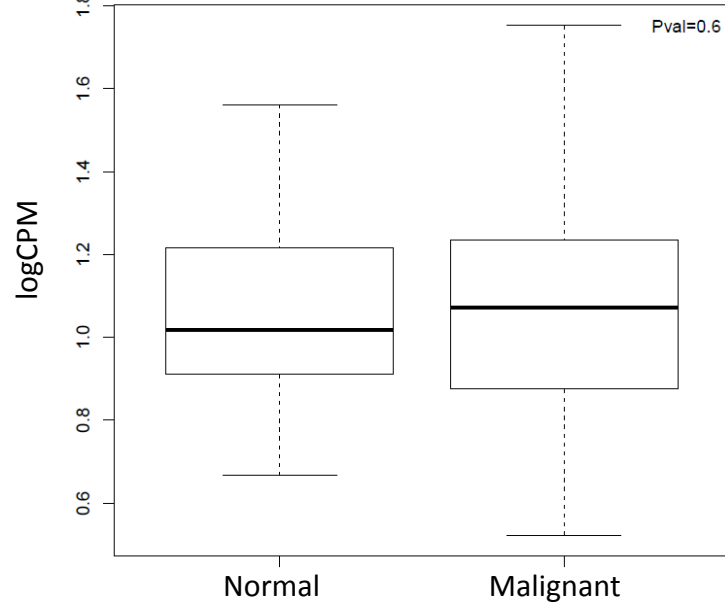

Supplement: Supplementary file 1 [file ijms-21-01386-s001.zip › 17_02_2020/Figure S3.pdf]

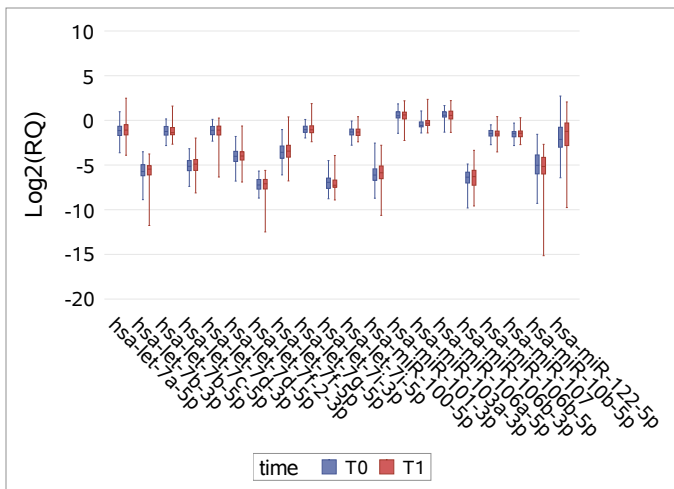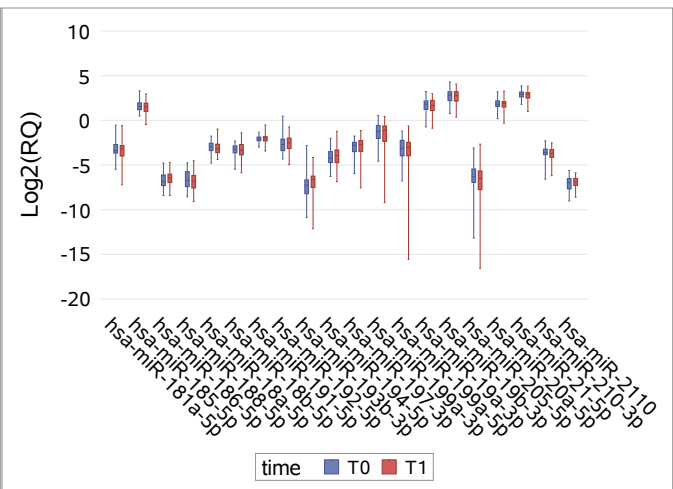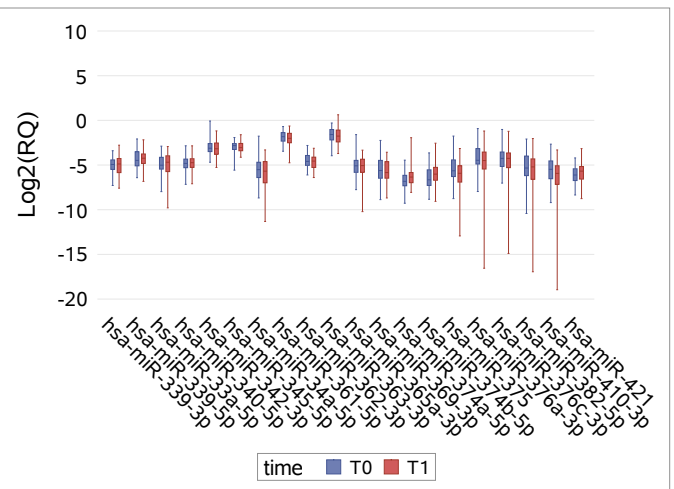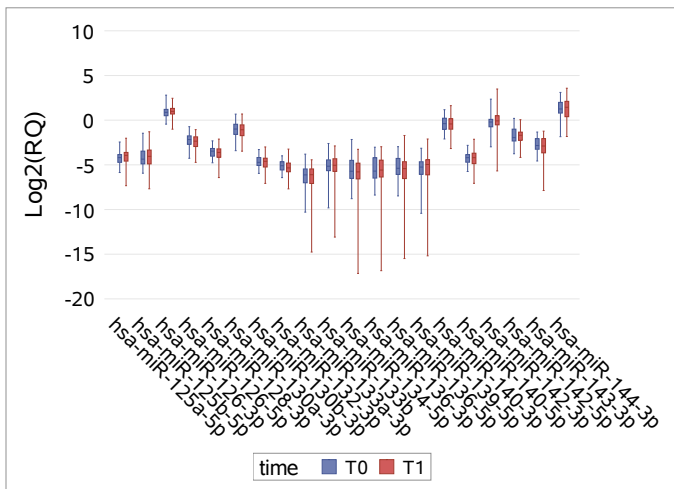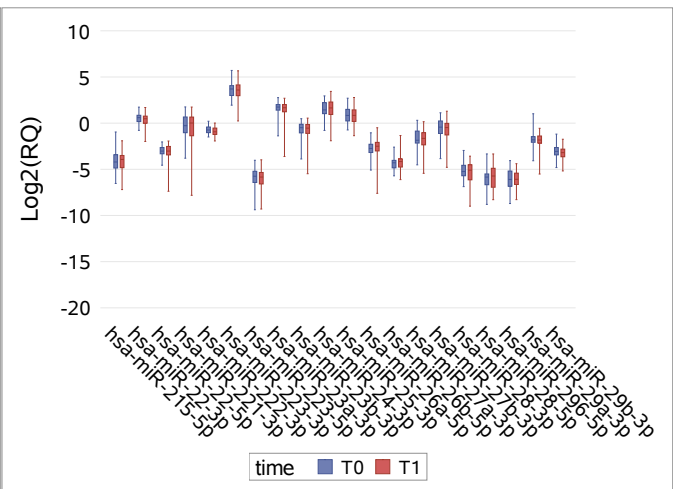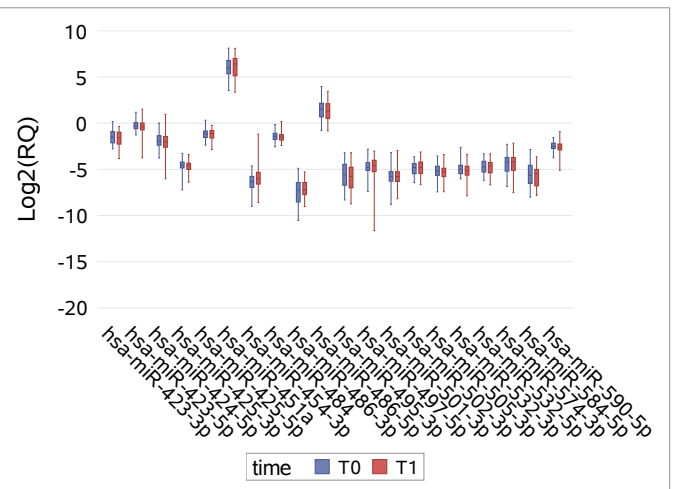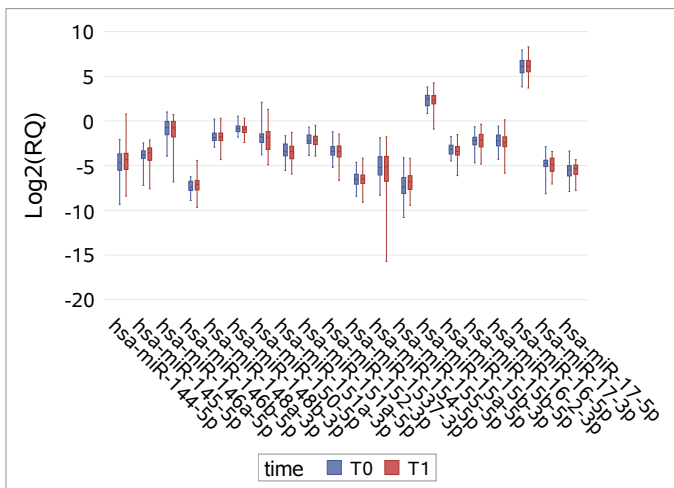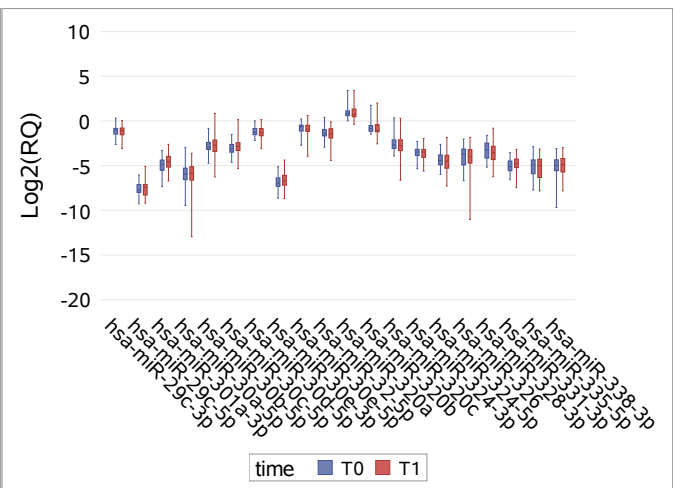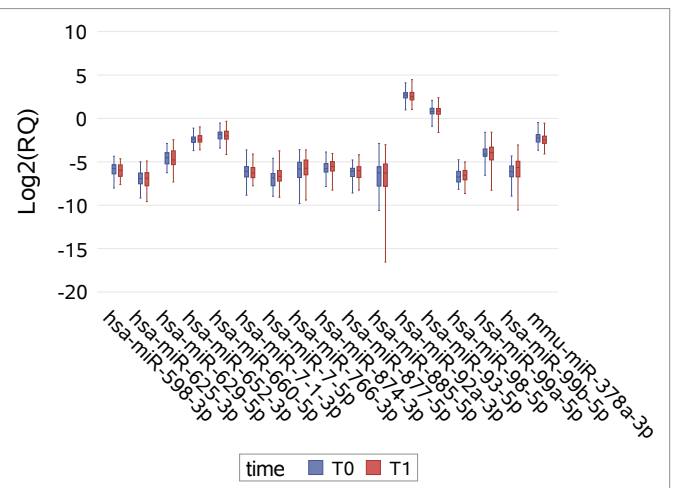

Supplement: Supplementary file 1 [file ijms-21-01386-s001.zip › 17_02_2020/FigureS1.pdf]

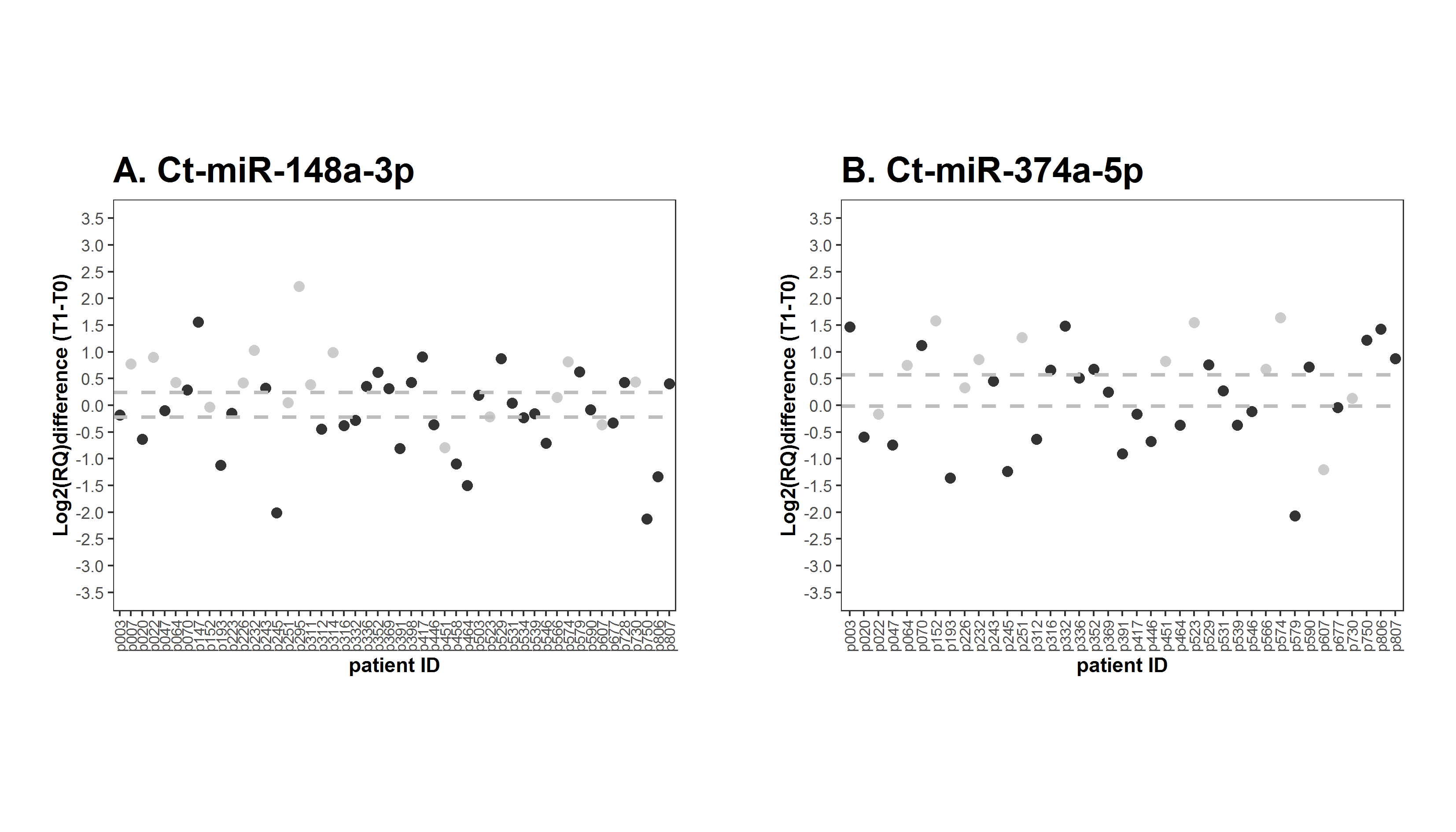

Supplement: Supplementary file 1 [file ijms-21-01386-s001.zip › 17_02_2020/FigureS2.tiff]

**Figure S1** Workflow of the study population

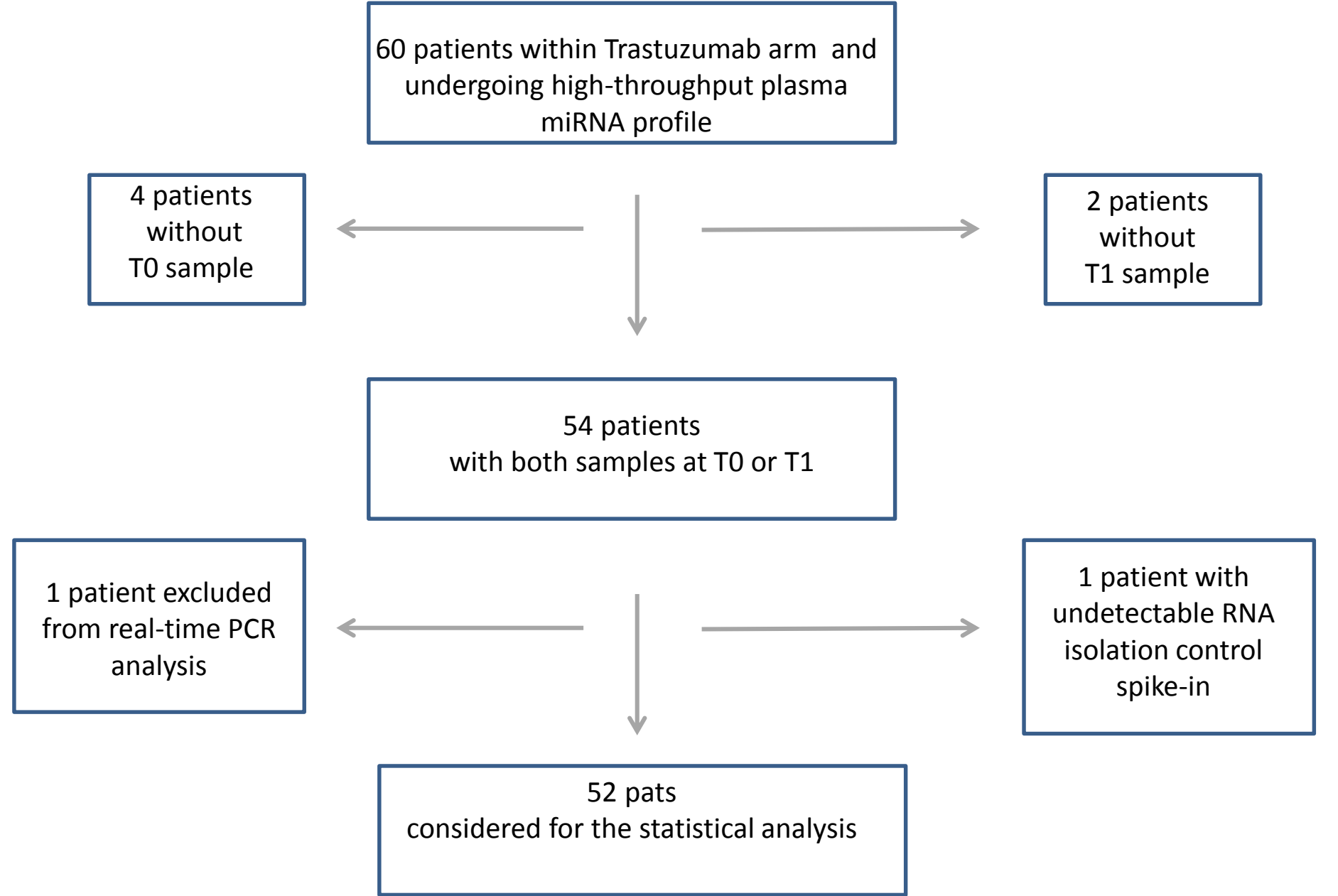

Supplement: Supplementary file 1 [file ijms-21-01386-s001.zip › 17_02_2020/FigureS4.pdf]
